# *In silico* testing of a multimaterial scaffold for mandibular reconstruction

**DOI:** 10.3389/fbioe.2025.1535756

**Published:** 2025-09-01

**Authors:** Pedro Rebolo, Vincenzo Orassi, Bruno Areias, Sara Checa, Nilza Ramião, Jaime Filipe Correia, Carsten Rendenbach, Renato Natal, Marco Parente

**Affiliations:** ^1^ INEGI - Institute of Science and Innovation in Mechanical and Industrial Engineering, Porto, Portugal; ^2^ Julius Wolff Institute, Berlin Institute of Health at Charité—Universitätsmedizin Berlin, Berlin, Germany; ^3^ Institute of Biomechanics, TUHH Hamburg University of Technology, Hamburg, Germany; ^4^ DEMec, Faculty of Engineering - University of Porto, Porto, Portugal

**Keywords:** finite element, bone regeneration, multimaterial scaffold, biomechanics, mechanobiology

## Abstract

**Introduction:**

Mandibular reconstruction following segmental resection is a challenging procedure. The implantation of scaffolds as an alternative for microsurgical free flaps appears as a promising strategy; however, there is still a lack of understanding of how such scaffolds should be designed to support bone regeneration. This study investigates the influence of scaffold design and its mechanical properties on the biomechanical conditions induced in mandibular reconstruction.

**Methods:**

A 3D finite element model of the human mandible was developed, and a large bone defect scenario was simulated, with physiological post-operative loading and boundary conditions. The large defect was bridged with a scaffold, supported by a titanium mesh, and stabilized with a load-bearing titanium fixation plate. To study the effect of the fixation device stiffness on the induced biomechanical conditions within the scaffold pores, two different materials were tested for the fixation device, namely, a Ti-6Al-4V titanium alloy and a polylactic acid (PLA). In addition, three different strut-based scaffold architectures were investigated with different strut orientations, while keeping the same strut diameter and similar overall porosity. Two types of material distributions through the scaffold were also studied. The first type was a hydrogel-based scaffold, whereas the second type was a multimaterial type where the scaffold was divided into three equal volume parts: in the center, a hydrogel material was employed, and in the extremities, a ceramic material. These combinations of two fixation materials and three scaffold architectures with two combination materials resulted in 12 experimental groups.

**Results and Discussion:**

No failure was predicted in the fixation devices for any of the configurations investigated. The PLA fixation device induced higher strains within the healing region than the titanium fixation device. Differences in scaffold architecture did not influence the strain levels within the healing region. Changes in the scaffold material distribution led to considerable differences in the mechanical strains within the scaffold pores. The multimaterial scaffold induced higher strains within the healing region than the only hydrogel scaffold, which might be beneficial to promote bone healing in the defect. Thus, a multimaterial scaffold seems to be able to provide a more suitable biomechanical environment to support bone regeneration, especially in large segmental defects. Future studies should focus on the mechanobiological optimization of the scaffold design and its fixation system in different clinical scenarios.

## 1 Introduction

Mandibular reconstruction due to segmental resections in patients with tumors or osteonecrosis is surgically challenging and associated with donor-site morbidity and postoperative complications, including non-union and plate exposure (Shaw et al., 2004; Evans et al., 1995; Isler et al., 2018; Rendenbach et al., 2018b; Rendenbach et al., 2018a; Catalá-Lehnen et al., 2012; Rendenbach et al., 2016; Rendenbach et al., 2018c; Steffen et al., 2023b; Steffen et al., 20232022). Segmental defects compromise the mechanics and function of the mandible, like basic masticatory functions and swallowing, speech articulation and phonation, and facial aesthetics (Orassi et al., 2021a; Rendenbach et al., 2018b). To maintain tongue mobility and restore the innervation of the affected area, a continuous bony mandible restoration is necessary (Rana et al., 2011).

The preferred treatment for mandibular reconstruction remains a fibula-free flap with a titanium fixation, since the stiffness of biodegradable plates and screws for the fixation in load-bearing situations is not sufficient (Steffen et al., 2020b; Steffen et al., 2020a). However, the procedure presents many limitations. Since each patient is unique, individualized planning and surgery are required, as no universally accepted protocol or set of guidelines exists for optimal preoperative, intraoperative, and postoperative flap management Rendenbach et al. (2018b), Kansy et al. (2014). Another drawback of this treatment is the compromised functionality of the donor site after free fibula flap harvesting, as the postoperative performance of the lower limb significantly declines, leading to a substantial impact on daily activities, equivalent to the loss of 7 years of health and fitness in a healthy individual (Rendenbach et al., 2016).

Alternative treatment methods for mandibular reconstruction are currently being researched. Modern technologies, such as 3D printing, are considered revolutionary ways of treating bone defects in the maxillofacial area (Zhang et al., 2019; Daniels et al., 2021; Tatullo et al., 2019). Biomedical research on mandibular reconstruction is nowadays focused on using innovative biomaterials with characteristics that can be tailored to promote healing and reconstruction of segmental bone defects (Khodakaram-Tafti et al., 2018; Liu et al., 2021).

Biomechanical conditions are known to significantly influence bone healing outcomes (Claes and Heigele, 1999; Orassi et al., 2021a; Orassi et al., 2021b; Ruf et al., 2022; Ruf et al., 2024). Computational studies have explored the mechanical conditions within scaffold pores and their relationship to the bone regeneration process (Rosa et al., 2023). In silico models for mandibular reconstruction have been developed to determine the biomechanical environment conducive to bone formation. However, these models have predominantly focused on the use of miniplates for fixation (Orassi et al., 2021a; Orassi et al., 2021b; Ruf et al., 2022; Ruf et al., 2024).

Several studies have investigated the potential of scaffolds for segmental mandibular reconstruction. *In vivo* animal studies using calcium phosphate-based ceramic scaffolds or hydroxyapatite and atelocollagen scaffolds have shown promising results (Vdoviaková et al., 2023). These studies demonstrated that such scaffolds provide an ideal environment for osteoblast transfer, adhesion, migration, and growth (Taha et al., 2023).

However, *in vivo* studies investigating the performance of scaffolds are very costly and time-consuming. As a result, *in silico* testing has become a more viable solution for evaluating scaffold performance. This type of testing allows for a systematic evaluation of different scaffold designs and materials. *In silico* models have been developed, validated, and employed to predict successful healing outcomes, providing a cost-effective and efficient alternative to *in vivo* studies Orassi et al. (2021b); Soufivand et al. (2020); Rosa et al. (2023).

The development of 3D printing has enabled the fabrication of scaffolds with sufficient porosity. 3D printed titanium scaffolds have demonstrated promising results in bone regeneration, successfully integrating the broken ends of bones. However, titanium use comes with several drawbacks, including the induction of metal imaging artifacts in computed tomography, cone beam computed tomography, and magnetic resonance imaging, which reduces diagnostic quality (Radzi et al., 2014; Rendenbach et al., 2018d; Demirturk Kocasarac et al., 2019). An alternative to this problem is the use of tissue scaffolds, which have shown desirable results (Ferguson et al., 2022; Cesarano III et al., 2005; Jo et al., 2017). Despite these advancements, the potential of a scaffold with different material sections for mandibular reconstruction has yet to be investigated.

This study aims to investigate the potential of a multimaterial scaffold to induce a favorable biomechanical environment for bone formation in a segmental mandibular defect. For this purpose, three scaffold architectures and two types of material distributions were used in this study.

## 2 Materials and methods

### 2.1 Scaffold morphology

The scaffold architecture was obtained using a combination of MATLAB code to generate a gcode file and Blender (Blender Foundation, Netherlands). The usage of MATLAB provided creative freedom in the architecture of the scaffold, so a custom scaffold could be more easily obtained. The scaffold morphology was aligned with the cut mandible surface, and its height was limited by the mandible’s height. This morphology was generated using a MATLAB code script where the. stl file of the scaffold outline was read, a virtual line intersected the scaffold. stl file, and the intersection points were written in a text file. The obtained gcode file was then imported into Blender to generate the 3D shape of the scaffold. Finally, a new. stl file was exported.

The scaffold porosity, P , was determined by Equation 1 (Soufivand et al., 2020), where $V_{C}$ is the volume of the solid shape that has the outline of the scaffold, and $V_{S}$ is the volume of the scaffold itself.

$$P=\left(1-\frac{V_{S}}{V_{C}}\right)\times 100$$ (1)

**FIGURE 1:**
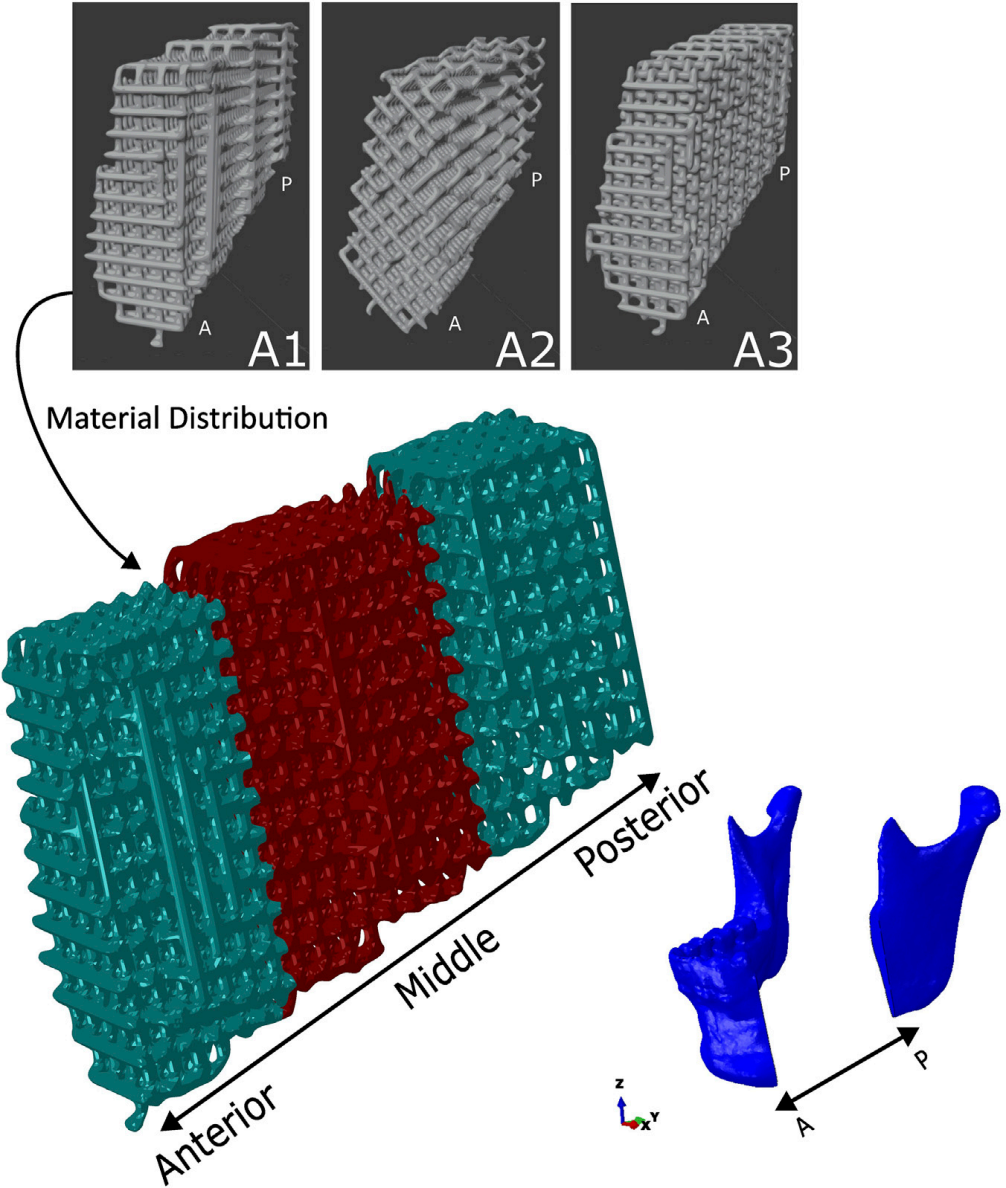
The three scaffold architectures: A1 (scaffold with orthogonal strut deposition), A2 (Scaffold with orthogonal strut deposition rotated 45°), A3 (Scaffold with orthogonal struts offset layer-by-layer to create a decentered pattern), and the material distribution in the scaffold using the A1 type architecture scaffold. The main scaffold model below illustrates the *in situ* positioning within the mandibular defect, segmented into anterior, middle, and posterior regions. The material distribution is colour-coded: ceramic (green) is located in the anterior and posterior ends, while hydrogel (red) is placed in the middle region. Mandibular anatomy is shown for orientation. Scaffold architecture and material placement are consistent across positions. P - Posterior; A- Anterior.

Three different scaffold architectures were developed for this study. The first architecture featured an orthogonal deposition of the struts (hereinafter A1). The second architecture also had an orthogonal deposition of the struts; however, the deposition was performed at a 45° angle (hereinafter A2). The third architecture was similar to the first, but the struts were offset from the corresponding struts in the previous layer, resulting in a decentering of the struts along the scaffold (hereinafter A3). Figure 1 illustrates the three scaffolds, and Table 1 presents the porosity values of the scaffolds.

**TABLE 1:** Values for the volumes and porosities of the scaffold architectures.

Scaffold	$V_{S}$ , ( ${\text{mm}}^{3}$ )	$V_{C}$ , ( ${\text{mm}}^{3}$ )	Porosity, (%)
A1	3889.0		65.7
A2	3589.9	11,340.6	68.3
A3	3766.7		66.8

### 2.2 Scaffold material distribution

As stated, the scaffold presented two types of material distribution. The first type of scaffold was composed entirely of hydrogel. The second type of scaffold was composed of two-thirds ceramic material and one-third hydrogel material. In this second type, the hydrogel material was distributed in the middle of the scaffold, while the ceramic material was distributed at the ends of the scaffold. The three regions—two ceramic and one hydrogel—each had approximately the same volume, as shown in Figure 1.

### 2.3 Finite element model

A cone-beam computed tomography (CBCT) scan of a human skull of a 20-year-old male individual was performed in axial mode, with a voxel size of 0.4 ${\text{mm}}^{3}$ (ProMax, Planmeca, Finland). Material segmentation of the fully dentate mandible was carried out in the software Amira 6.0.1 (Zuse Institute Berlin, Germany), labeling cortical and trabecular bone tissues. After the segmentation, a primary mesh was assigned to the segmented bones. The bones were then exported as a. stl object to the 3D-CAD software SolidWorks 2023 to perform a virtual resection of the mandible. This virtual resection was done on the left side of the mandible, going from the mandibular angle to the left canine. The resulting gap had a length of 4 cm. Holes in the mandible were also made to hold the fixation device on the mandible. The obtained mandible section was then imported to Abaqus/CAE (Dassault Systèmes Simulia Corp., United States) and meshed with linear tetrahedral elements (C3D4).

**FIGURE 2:**
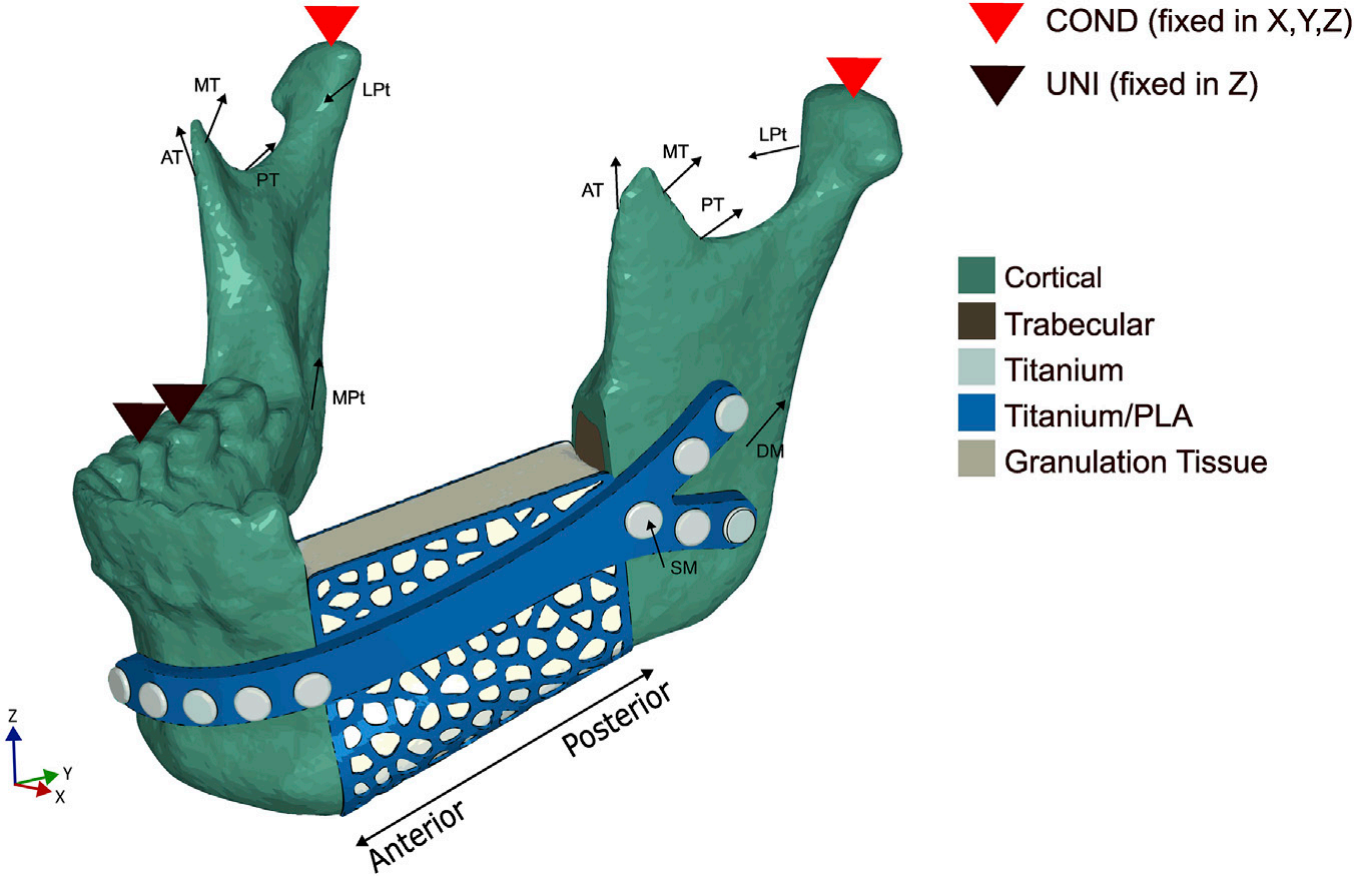
Loading and boundary conditions used in the numerical model during the unilateral (UNI) clenching. The bite force was simulated by restraining the vertical displacement at the occlusion and the condyles (COND) in the 6 degrees of freedom. SM - Superficial masseter; DM - Deep masseter; AT - Anterior temporalis; MT - Medial temporalis; PT - Posterior temporalis; MPt - Medial pterygoid; LPt - Lateral pterygoid.

The fixation device can be divided into two sections, the plate and the cage. The plate was designed using the 3D-CAD software SolidWorks 2023 (Dassault Systèmes, France). One end of the fixation device has a Y-shape branching structure, while the other end has a typical linear design. The plate had a 3 mm thickness, with the screw hole having a 2.7 mm diameter. Attached to the plate was a cage, with a thickness of 0.5 mm, that was partially wrapped around the scaffold to give mechanical support and keep the scaffold in place during bone regeneration. The patient-specific cage was designed using the outer surface of the missing bone piece. The cage was then imported to Meshlab, where the holes in the structure were created using a Poisson Disk Sampling. These holes allow the passage of nutrients as well as reducing the weight in the structure. Both sections were then merged using Meshmixer (Autodesk, United States), and a 3D linear triangular mesh, with an edge length of 0.5 mm, was created. This model was then imported into the commercial software Abaqus/CAE (Dassault Systèmes Simulia Corp., United States) and the triangular mesh was converted into a linear tetrahedral mesh (element type C3D4). The entire fixation device is shown in Figure 2 in blue.

Bicortical screws with various lengths (11 mm, 13 mm, 15 mm, and 17 mm) were used to fix the fixation device to the mandible. Tie constraints were applied between the fixation device and screws and between the mandibular bone tissues and screws (Orassi et al., 2021b). Since the expected displacements of the scaffold are very small, tie constraints were applied between the scaffold and the trabecular and cortical bones in order to simplify the numerical model and to reduce computational cost.

### 2.4 Loading and boundary conditions

A unilateral clenching was chosen as the only task to be tested, since several studies (Korioth et al., 1992; Lakatos et al., 2014; Lovald et al., 2009) reported that a contralateral molar occlusion induced the highest amount of mechanical solicitation and, as such, represented a critical clenching task. For this particular study, a right unilateral clenching was simulated. By following the method used by Orassi et al. (2021b), the vertical displacement was restrained on the first molar-second premolar teeth group. The condyles were also assumed to be locked in the glenoid fossa and therefore restrained in all degrees of freedom.

Based on the study performed by Steffen et al. (2023a), the maximum mean average post-operative bite force for patients who underwent mandibular reconstruction was 131.5 N. These post-operative loading conditions were applied by simulating the contraction of the superficial masseter (SM), deep masseter (DM), anterior temporalis (AT), medial temporalis (MT), posterior temporalis (PT), medial pterygoid (MPt), and inferior lateral pterygoid (LPt) muscles. The directions of the force vectors were based on Korioth et al. (1992). All considered loading and boundary conditions are shown in Figure 2. The magnitude of the muscular forces was reduced to 20% (Orassi et al., 2021b) of the maximum muscle forces reported by Nelson (1986), except for the left superficial masseter and left deep masseter, which were reduced to 10% to simulate the partial detachment of the muscle in the post-operative scenario, resulting in a bite force of 115 N. The post-operative muscle forces, as well as the directions for the force vectors, are depicted in Table 2.

**TABLE 2:** Loads and direction cosines of muscle groups for unilateral clenching in the human mandible.

Mandibular Muscle Group	Maximum Muscle Force (N)	Post-Operative Muscle Force (N)	Direction Cosine			
			X		Y	Z
			Right	Left		
Superfical masseter	190.4	19.04/38.08	-0.207	0.207	-0.419	0.884
Deep masseter	81.6	8.16/16.32	-0.546	0.546	0.358	0.758
Anterior temporalis	158.0	31.60	-0.149	0.149	-0.044	0.988
Medial temporalis	95.6	19.12	-0.222	0.222	0.500	0.837
Posterior temporalis	75.6	15.12	-0.208	0.208	0.855	0.474
Medial pterygoid	174.8	34.96	0.486	-0.486	-0.373	0.791
Lateral pterygoid	66.9	13.38	0.630	-0.630	-0.757	-0.174

### 2.5 Material properties

Isotropic material properties were assigned to all materials. For the fixation device, i.e., the plate and the cage, two types of material were assigned: a titanium alloy and PLA. The yield strength of the titanium alloy was assumed to be 880 MPa, and for the PLA, the yield strength was assumed to be 70 MPa. The elastic modulus of the cortical and trabecular bone was assumed to be 15,000 MPa and 300 MPa, respectively, and the elastic modulus of the titanium and PLA materials was assumed to be 110,000 MPa and 3500 MPa, respectively (Orassi et al., 2021b; Saini et al., 2020). Isotropic material properties were also assigned to the tissue volume, modeled as granulation tissue, which represents the hematoma that forms after the resection (Checa et al., 2011).

The development of the hydrogel and calcium phosphate (CaP) platelet lysate composites used in the scaffold was developed by employing established methods from prior studies (Kimsal et al., 2011; Torres et al., 2016). The development of the hydrogel and ceramic material used in the scaffold was done by Universidade de Aveiro (UAVR). The corresponding material properties were obtained through compressive tests.

All of the material properties used in the numerical model are present in Table 3.

**TABLE 3:** Isotropic material properties assigned to the mandibular bone tissues, fracture volume, implants and scaffolds.

Material	Elastic modulus, E (MPa)	Poisson’s ratio, (−)	References
Cortical	15,000	0.30	Saini et al. (2020)
Trabecular	300	0.30	Orassi et al. (2021b)
Titanium	110,000	0.34	Lovald et al. (2009)
PLA	3500	0.36	Torres et al. (2015)
Granulation Tissue	0.2	0.167	Perier-Metz et al. (2022)
Hydrogel	25E-03	0.45	[-]
Ceramic	150	0.3	[-]

### 2.6 Model summary

**FIGURE 3:**
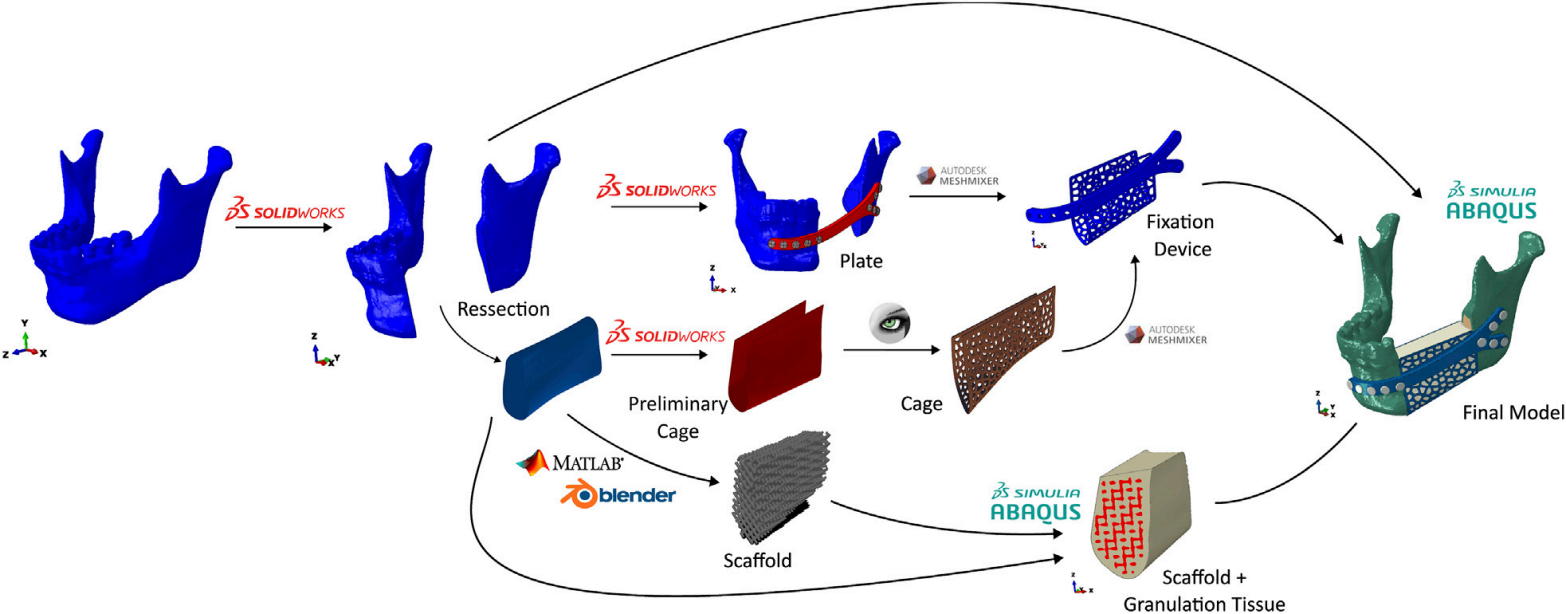
Numerical model development flowchart.

A summary of the numerical model development is shown in Figure 3. The acquired mandible was imported into SolidWorks 2023 where a virtual resection was performed. The fixation device plate was also done using SolidWorks by following the curvature of the mandible. The cage was obtained by using the removed section of the mandible. Afterwards, an. stl file of the cage was imported into Meshlab to create the holes. Both the plate and cage were then merged using Meshmixer to create the fixation device.

The scaffold was done by a combination of MATLAB and Blender. The. stl file of the removed section was imported into MATLAB where a gcode file was created. This gcode file was then imported into Blender, where the. stl file of the scaffold was created. In order to emulate the granulation tissue, the generated scaffold and the base. stl file were imported into Abaqus/CAE and merged.

Finally, all of the elements were combined in Abaqus to create the numerical model.

### 2.7 Mesh convergence study

A mesh convergence study was performed on the healing region (granulation tissue + scaffold), since it is the main focus of this study. Three different mesh sizes were used in this convergence study. Mesh A is the finest mesh, with a total of 7,180,810 elements, mesh B has 4,843,5811 elements, and mesh C, the coarsest mesh, with 4,371,508 elements. Specifically, the elastic modulus of the scaffold was chosen to be 16 kPa, and the Poisson’s ratio to be 0.3. These values were based on earlier tests of the hydrogel material. A simple displacement of 1 mm was applied to the top (Posterior) of the scaffold, and the base (Anterior) of the scaffold was pinned. The von Mises stresses and the strains of the scaffold and granulation tissue were compared between the three meshes.

The average aspect ratio for meshes A, B, and C is, respectively, 1.70, 1.68, and 1.65. The number of elements that have an aspect ratio greater than 5 is less than 0.02% of the total number of elements for all three meshes. The skewness for mesh A, B, and C is, respectively, 0.37, 0.36, and 0.34. A simple linear tetrahedral mesh was used in the granulation and scaffold region.

Mesh B resulted in stresses and strains comparable to the finest mesh (mesh A), with a relative error of 1% regarding the von Mises stresses and a relative error of 2% for the strain. Therefore, mesh B was chosen for the remainder of the study.

## 3 Results

### 3.1 von mises stresses in the fixation system

**FIGURE 4:**
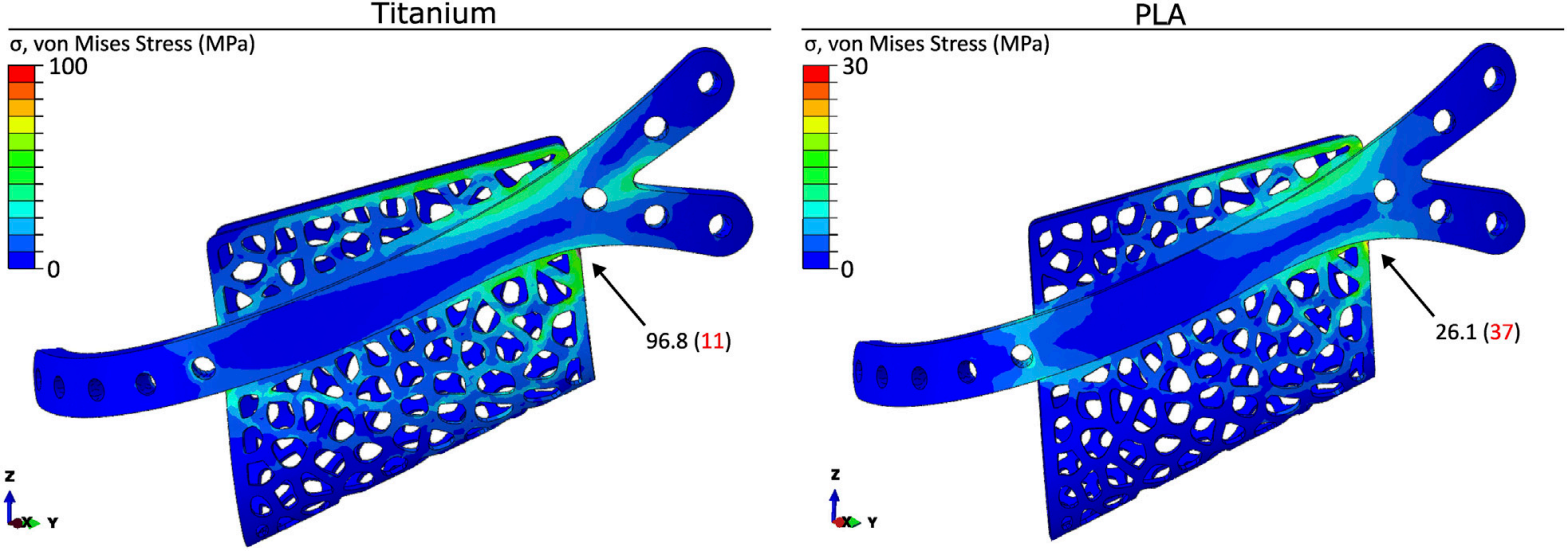
Distribution and peak values (in MPa) of the von Mises stress in the titanium and PLA fixation devices. Stress concentrations are visualised using a colour gradient, with high stress regions in red and low stress regions in blue. The location of the peak value (in MPa) is indicated in the figure. The values in brackets represents the peak stress as a percentage of the yield strength for titanium and PLA.

The von Mises stress distribution and peak values for the titanium alloy Ti-6Al-4V and for the PLA polymer are shown in Figure 4. For both cases, the maximum stress was lower than the corresponding yield stress, which meant that both materials worked in the elastic region. The fixation device made of titanium showed maximum von Mises stress values around 11% of the titanium yield strength, while in the PLA fixation device, the maximum von Mises stress was around 37% of the PLA yield strength. These results were consistent for the different architecture and material models used in the scaffold.

### 3.2 Scaffold material with Ti-6Al-4V fixation

**FIGURE 5:**
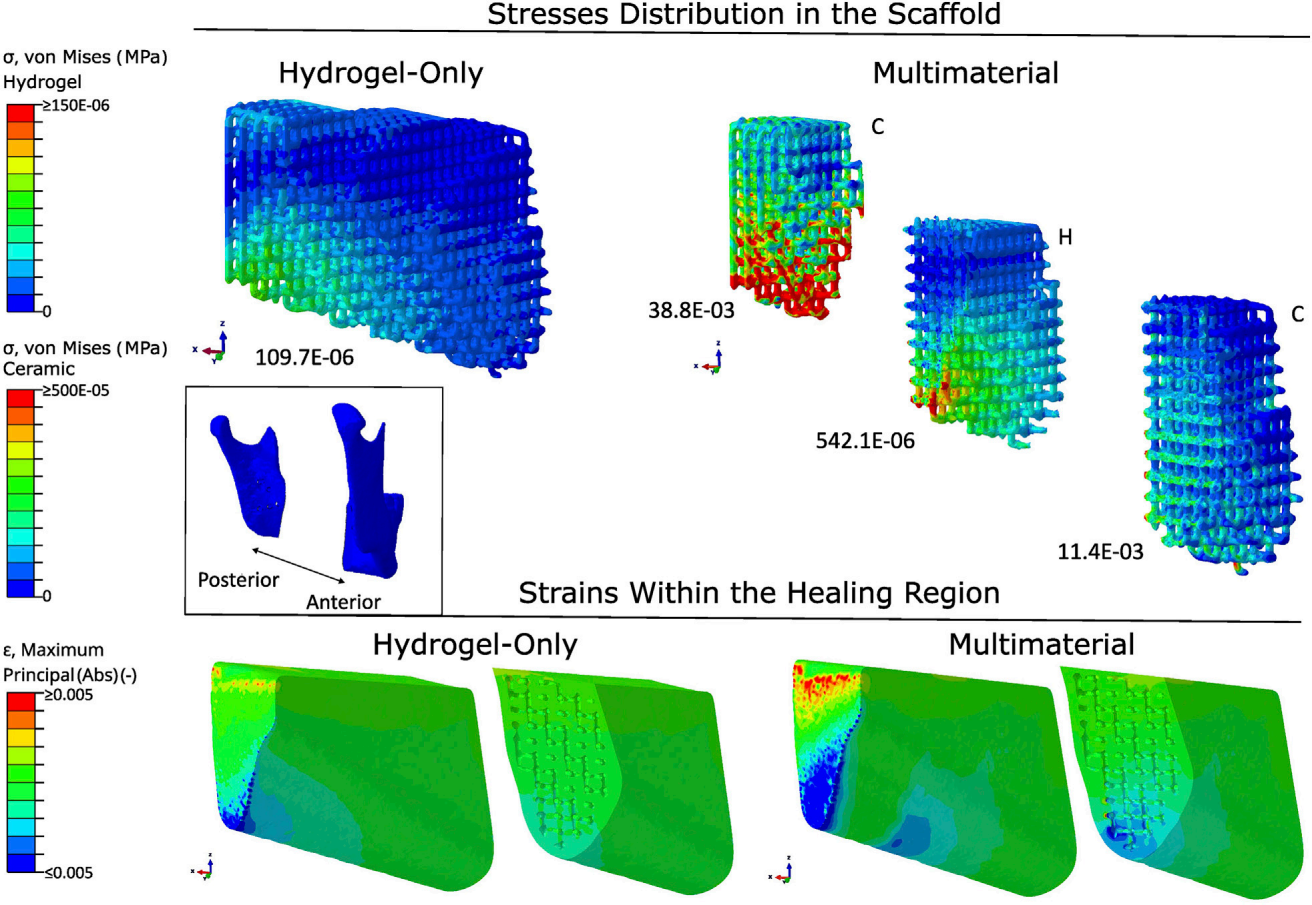
Stress distribution in scaffolds and strain patterns within the healing region, comparing hydrogel-only and multimaterial scaffolds with PLA fixation and A1 architecture. Stress concentrations are visualised using a colour gradient, with high stress regions in red and low stress regions in blue. Peak stress values are reported below each scaffold (in MPa). Strain mapping within the healing region distinguishes between compressive strains (blue) and tensile strains (red). The scaffold orientation and healing region are aligned to the anatomical position of the mandible. Material abbreviations: C, Ceramic; H, Hydrogel.

The distribution of the von Mises stresses and the absolute strains in both types of scaffold material using the titanium fixation and the A1 scaffold architecture is shown in Figure 5. In the hydrogel-only scaffold, the stresses were concentrated at the posterior region of the scaffold, with the maximum magnitude being 109.7E-06 MPa. With the usage of a multimaterial scaffold, the stresses were more distributed throughout the scaffold. The maximum stress magnitude was 38.8E-03 MPa, and in the middle region of the scaffold, where the scaffold is made up of hydrogel, the stress magnitude reached 542.1E-06 MPa.

**FIGURE 6:**
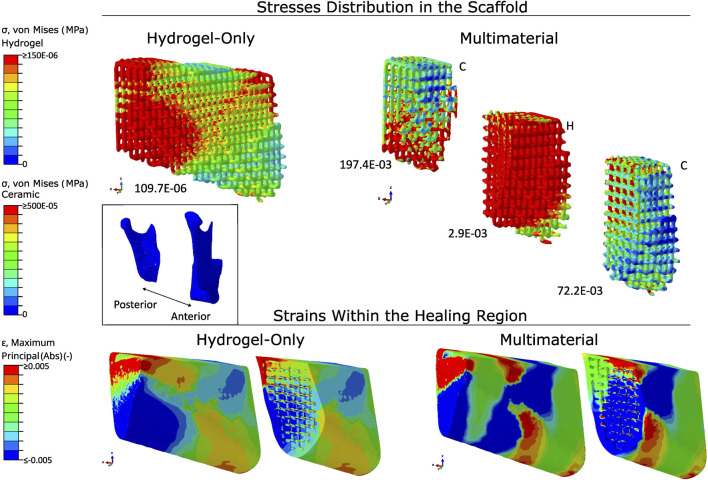
Stress distribution in scaffolds and strain patterns within the healing region, comparing hydrogel-only and multimaterial scaffolds with titanium fixation and A1 architecture. Stress concentrations are visualised using a colour gradient, with high stress regions in red and low stress regions in blue. Peak stress values are reported below each scaffold (in MPa). Strain mapping within the healing region distinguishes between compressive strains (blue) and tensile strains (red). The scaffold orientation and healing region are aligned to the anatomical position of the mandible. Material abbreviations: C, Ceramic; H, Hydrogel.

The absolute strains within the healing region are also present in Figure 6. In both cases, the strains were mostly located in the posterior region. There were also some strains in the middle region with the use of a multimaterial scaffold. With the use of a hydrogel scaffold, the induced absolute mechanical strains were between 0.02% and 0.07%, with the median being 0.04%.

### 3.3 Scaffold material with PLA fixation

**FIGURE 7:**
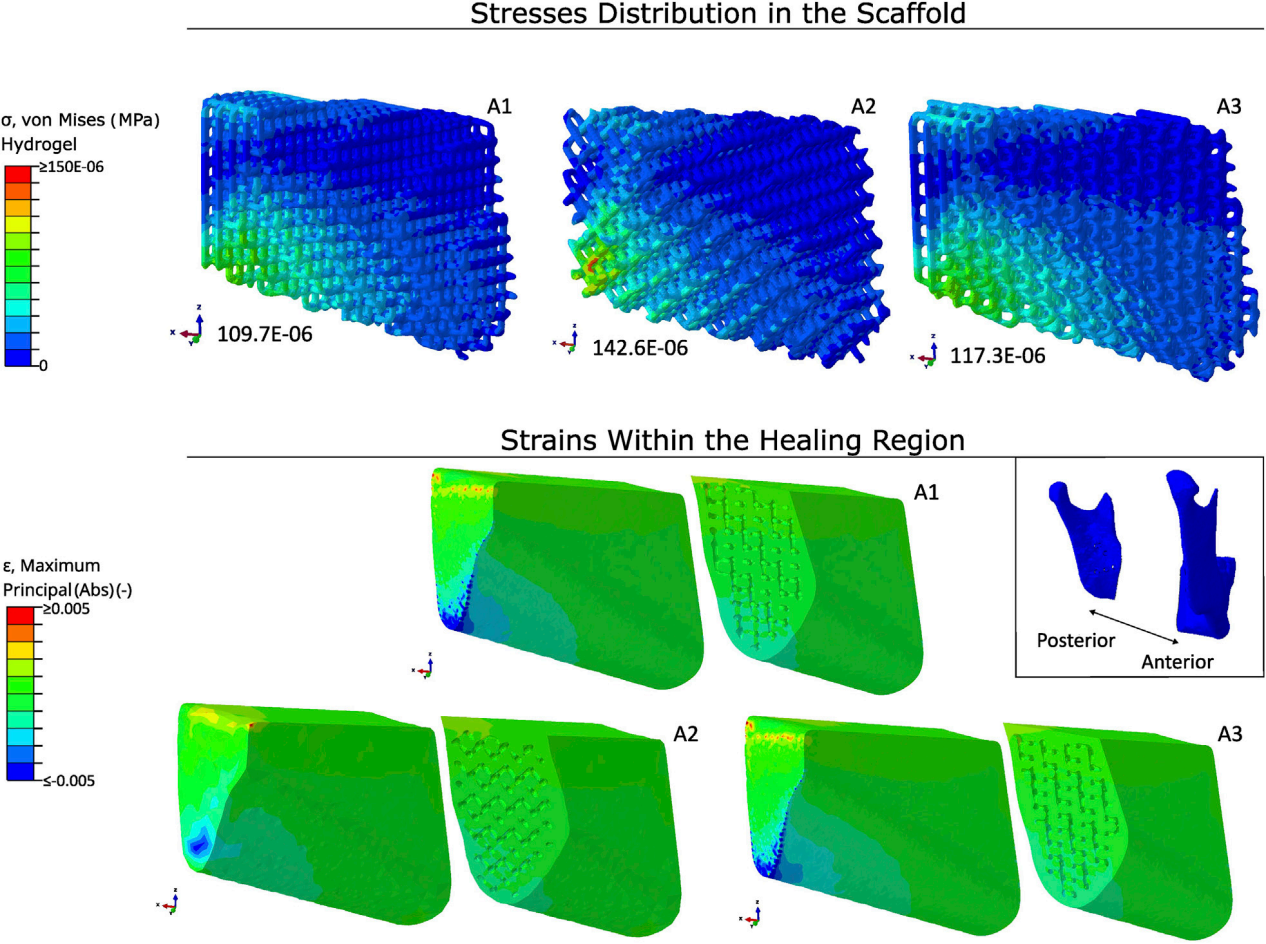
Stress distribution in scaffolds and strain patterns within the healing region using hydrogel-only scaffolds with titanium fixation. Stress concentrations are visualised using a colour gradient, with high stress regions in red and low stress regions in blue. Peak stress values are reported below each scaffold (in MPa). Strain mapping within the healing region distinguishes between compressive strains (blue) and tensile strains (red). The scaffold orientation and healing region are aligned to the anatomical position of the mandible. Type of architecture is indicated on the top right of each scaffold.

The distribution of the von Mises stresses and the absolute strains in both types of scaffold material using the PLA fixation and the A1 scaffold architecture is shown in Figure 7. In the hydrogel-only scaffold, the stresses were concentrated at the posterior region and the top of the anterior region of the scaffold, with the maximum magnitude being 689.1E-06 MPa. In the case of using a multimaterial scaffold, the maximum stress magnitude was 197.4E-03 MPa and in the middle region of the scaffold, where the scaffold is made up of hydrogel, the stress magnitude reached 2.9E-03 MPa.

The absolute strains within the healing region are, once more, present in Figure 5. The strains were located in the posterior and middle of the healing region. The compressive and tensile strains were higher in the inferior and top regions, respectively, due to a combination of bending and torsional movements. There was a switch in the position of the compressive and tensile strains within the healing region at the middle and anterior regions, with the compressive strains being located in the top region and the tensile strains being located in the inferior region. With the use of a hydrogel scaffold, the induced absolute mechanical strains were between 0.2% and 0.5%, with the median being 0.3%. The use of a multimaterial increased the strains in the middle of the healing region, where the hydrogel part of the multimaterial scaffold was located. The absolute strains in this hydrogel part were of 0.7%.

### 3.4 Scaffold architecture with Ti-6Al-4V fixation

In Figure 7, the von Mises stresses in a hydrogel-only scaffold and the absolute strains within the healing region are represented using different scaffold architectures employing a titanium fixation. The stresses were mainly concentrated at the lower posterior region. The peak values for the von Mises stresses in architectures A1, A2, and A3 were 109.7E-06 MPa, 142.6E-06 MPa, and 117.3E-06 MPa, respectively.

The distribution of the absolute strains were similar in all three architectures, with the strains being located at the lower posterior region of the healing region. The median values for the strains within the healing region were, again, similar in the three architectures, being around 0.04%.

### 3.5 Scaffold architecture with PLA fixation

**FIGURE 8:**
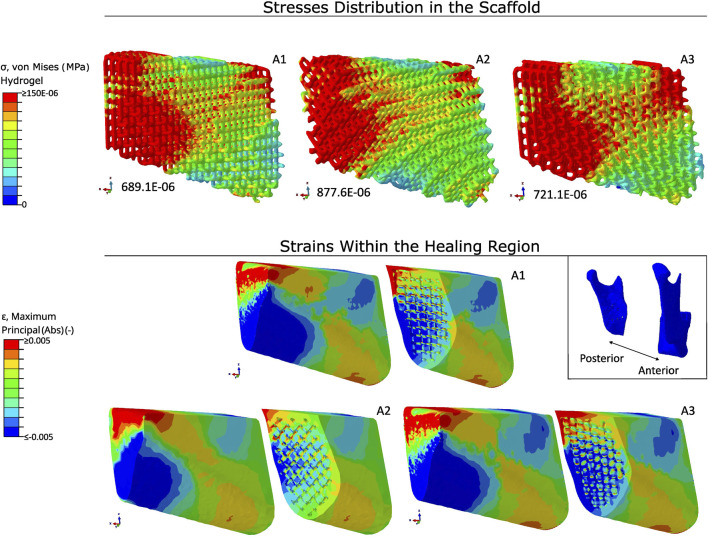
Stress distribution in scaffolds and strain patterns within the healing region using hydrogel-only scaffolds with PLA fixation. Stress concentrations are visualised using a colour gradient, with high stress regions in red and low stress regions in blue. Peak stress values are reported below each scaffold (in MPa). Strain mapping within the healing region distinguishes between compressive strains (blue) and tensile strains (red). The scaffold orientation and healing region are aligned to the anatomical position of the mandible. Type of architecture is indicated on the top right of each scaffold.

In Figure 8, the von Mises stresses in a hydrogel-only scaffold and the absolute strains within the healing region are represented using different scaffold architectures employing a PLA fixation. The stresses were concentrated at the posterior region and at the anterior region of the scaffolds. The peak values for the von Mises stresses in architectures A1, A2, and A3 were 689.1E −06 MPa, 877.6E −06 MPa, and 721. E −06 MPa, respectively.

The distribution of the absolute strains were similar in all three architectures. As stated in the scaffolds material with PLA fixation section, the compressive and tensile strains were higher in the inferior and top regions, respectively, due to a combination of bending and torsional movements. There was a switch in the position of the compressive and tensile strains within the healing region at the middle and anterior regions, with the compressive strains being located in the top region and the tensile strains being located in the inferior region. A1 and A3 had similar values for the absolute principal strains, with median values around 0.31%. The absolute principal strains for A2 had slightly lower strains, with a median absolute principal strain of 0.26%.

## 4 Discussion

The study aimed to evaluate the performance of a multimaterial scaffold for treating a mandibular resection from a biomechanical perspective. Titanium scaffolds have been commonly used for large bone defects Perier-Metz et al. (2022); however, these scaffolds are not biodegradable, meaning they remain in place after healing. To address this limitation, biodegradable materials were explored. Calcium-phosphate-based biomaterials, well-established in maxillofacial surgery, offer advantages in reconstructive and regenerative medicine (Vdoviaková et al., 2023). The materials used were developed by the Universidade de Aveiro (UAVR) for this project. The material distribution within the multimaterial scaffold was designed to position the ceramic material in contact with the existing bone, enhancing the transmission of mechanical strains throughout the healing region. The multimaterial scaffold showed promise in increasing strains within the inner scaffold pores, which has been associated with promoting bone regeneration (Rendenbach et al., 2019; Pobloth et al., 2018). The ceramic material in the multimaterial scaffold appeared to significantly increase mechanical strains in the region containing the hydrogel section; however, due to its stiffness, it generated lower mechanical strains in the region where the ceramic section was located.

The mandibular muscle forces were reduced consistently to achieve a post-operative bite force typically observed after surgery, to simulate the corresponding biomechanical conditions (Steffen et al., 2023a). By employing the method used by Orassi et al. (2021b) and reducing the maximum muscle force to 20%, the obtained bite force exceeded the force reported by Steffen et al. (2023a). Since the mandibular resection removed a large bone section from the left side of the mandible, a reduction of the muscle force to 10% in the superficial masseter (SM) and the deep masseter (DM) muscle groups was applied. With this reduction, the bite force achieved through the simulation was 115 N, which achieved results comparable to the results obtained from Steffen et al. (2023a), where the maximum post-operative bite was 131.5 N.

The peak von Mises stresses within the fixation device indicated that both fixation materials were capable of sustaining the loads across all six scaffold designs without structural failure. The PLA fixation device showed a less favourable ratio between peak von Mises stresses and yield strength compared to the titanium alloy fixation device. However, the stresses observed in PLA remained within the elastic region under a post-operative bite force scenario. The predicted strains within the healing region indicated that the titanium fixation device provided higher fixation stiffness, resulting in significantly lower strains within the healing region than with the PLA fixation, regardless of the scaffold used, which could impair bone regeneration. This limitation could potentially be addressed by reducing the plate thickness, allowing the reduced stiffness to increase mechanical strains within the healing region.

Scaffolds emerged as a promising strategy for treating large segmental mandibular defects; however, the influence of scaffold design on the biomechanical conditions induced within the healing region remained unknown. The biomechanical environment created by the scaffold was crucial, as it needed to generate beneficial mechanical strains within the healing region to promote bone formation (Rendenbach et al., 2019; Pobloth et al., 2018). Mechanical strains have been shown to influence bone regeneration within the healing region, with strain levels below 15% (ε<15%) reported as beneficial in long bones (Claes and Heigele, 1999; Pobloth et al., 2018; Duda et al., 2023). However, it was also noted that mechanical strains in large defects tended to be lower (Mehta et al., 2012; Pobloth et al., 2018).

Considering the impact of scaffold materials on strain distribution within the healing region, the use of a multimaterial scaffold improved strains within this region. With the PLA fixation device (since the strains within the healing region were low when using a titanium fixation), the median mechanical strain for a hydrogel-only scaffold within the healing region was around 0.3%. When using the multimaterial scaffold, the mechanical strains in the hydrogel region increased to approximately 0.7%. A previous study reported average strain values between 0.07% and 0.61% within the interosseous gap (Ruf et al., 2022); however, that study used a bite force of 45 N, while the present study applied a bite force of 115 N. Another study suggested that mechanical strains ranging from 0.23% to 0.6% might be beneficial for endochondral and intramembranous ossification (Pobloth et al., 2018). Furthermore, in the observed differences of absolute mechanical strains throughout the healing region, strain magnitudes were more prominent in the posterior and middle regions, while strains in the anterior region were lower. This pattern was consistent with findings by (Orassi et al., 2021b) and computer model predictions (Tams et al., 1999; Kimsal et al., 2011; Lovald et al., 2009), which indicated that mandibular bone healing may occur at higher mechanical strains in the posterior region, where mandibular muscle forces are applied, and at lower strains in the anterior region.

Three different architectural types were employed to assess whether differences in orientation affected the mechanical strains observed within the healing region. According to the literature, scaffolds with a porosity between 50% and 80% are beneficial for mimicking the extracellular matrix of native bone (Liang et al., 2019). The three architectures had similar porosity values, ranging between 66% and 68%, as shown in Table 1, which aligns with the porosity values reported by Liang et al. (2019). Based on the results, there were no notable differences in strain values among the different architectures. Likewise, there was no noticeable difference in the von Mises stress distribution across the three scaffold architectures. The distribution and magnitude of von Mises stresses were higher in the posterior region of the scaffold, which was closer to the applied mandibular forces. In the hydrogel-only scaffold, the stress distribution was very localized in the posterior region, with a relatively low von Mises stress magnitude. When ceramic material was introduced into the posterior and anterior regions of the scaffold, the distribution and magnitude of the von Mises stresses improved, particularly in the middle and anterior regions, whereas in the hydrogel scaffold, stresses were practically non-existent.

This study presented some limitations, one of which was the use of isotropic material properties for the cortical bone to simplify the model, since the main focus of the study was the impact of type and material distribution of the scaffold within the healing region. In future studies, cortical bone should be segmented following the methods of Schwartz-Dabney and Dechow (2003) and Lovald et al. (2009) and assigned orthotropic material properties to more accurately represent the mandibular bone. Although not present in this study, previous works have tested the influence of orthotropic properties of the cortical bone and found that there were no considerable changes in the mechanical strains (Orassi et al., 2021a). So it is expected that the mechanical strains using the orthotropic material properties would not differ much from the mechanical strains in this study.

Soft tissue, such as the periodontal ligament (PDL), was also omitted in this study. A study done by Panagiotopoulou et al. (2011) using a macaque mandible found that PDL can be excluded from finite element models if the area of interest is away from the alveolar region. However, this has been disputed. A study from Gröning et al. (2011) recommended that PDL should be included in finite element studies of the masticatory apparatus. By using a bite force on the right first molar, a similar approach to this work, the study reported that the model suffered overall greater deformation when using PDL. The maximum and minimum strains increased in the balancing side of the mandible, i.e., the opposite side of the biting point. That increase could reach around 500με . The PDL Young’s modulus can also affect the strains. With a modulus of 100 MPa, the mean strains increased up to 60%, and with a modulus of 1 and 0.01 MPa, the mean strains increased around 100%–200% when compared with a model where PDL was not used. Future studies should include PDL in the model, since the addition of this soft tissue might provide better results in terms of mechanical strains within the healing region.

Another limitation was that only a unilateral biting task was evaluated. Nonetheless, the unilateral biting task was chosen as the primary task, as it has been demonstrated to be critical for primary fixation stability (Ruf et al., 2022; Orassi et al., 2021a; Korioth et al., 1992; Lovald et al., 2009). Additionally, the tested scaffolds had similar porosity since the objective was to investigate if the architecture of the scaffold had a big influence on the biomechanical environment in the healing region; future studies should investigate various porosity levels and strut diameters. Tie constraints were also applied between the scaffold and the trabecular and cortical bone. This was performed to reduce the computational cost of the model. Future studies should consider a sliding contact interaction so that a better representation is achieved through the finite element model. However, the friction coefficient between the scaffold material and the interface of the trabecular and cortical bone should be known.

In summary, this study investigated the biomechanical performance of a multimaterial scaffold for the treatment of a mandibular bone defect. From a biomechanical perspective, the multimaterial scaffold showed promise as a strategy to increase intra-scaffold strain, potentially enhancing bone regeneration. The ceramic material in the multimaterial scaffold caused a notable increase in mechanical strains in the region where the hydrogel scaffold section was located; however, due to its higher stiffness, it produced lower mechanical strains in the ceramic region. To improve strain distribution further, the porosity of the ceramic scaffold could be increased to reduce stiffness, allowing for increased mechanical strains. The computer model developed in this study supported the development of treatment strategies for mandibular reconstruction.

## Data Availability

The original contributions presented in the study are included in the article, further inquiries can be directed to the corresponding author.
